# Aberrant Gut Microbiome Contributes to Intestinal Oxidative Stress, Barrier Dysfunction, Inflammation and Systemic Autoimmune Responses in MRL/lpr Mice

**DOI:** 10.3389/fimmu.2021.651191

**Published:** 2021-04-12

**Authors:** Hui Wang, Gangduo Wang, Nivedita Banerjee, Yuejin Liang, Xiaotang Du, Paul J. Boor, Kristi L. Hoffman, M. Firoze Khan

**Affiliations:** ^1^ Department of Pathology, University of Texas Medical Branch, Galveston, TX, United States; ^2^ Department of Microbiology and Immunology, University of Texas Medical Branch, Galveston, TX, United States; ^3^ Alkek Center for Metagenomics and Microbiome Research, Department of Molecular Virology and Microbiology, Baylor College of Medicine, Houston, TX, United States

**Keywords:** microbiome, oxidative stress, permeability, inflammation, autoimmunity

## Abstract

Microbiome composition and function have been implicated as contributing factors in the pathogenesis of autoimmune diseases (ADs), including systemic lupus erythematosus (SLE), rheumatoid arthritis and autoimmune hepatitis (AIH). Furthermore, dysbiosis of gut microbiome is associated with impaired barrier function and mucosal immune dysregulation. However, mechanisms by which gut microbiome contributes to the ADs and whether antioxidant treatment can restore gut homeostasis and ameliorate the disease outcome are not known. This study was, therefore, focused on examining the involvement of gut microbiome and host responses in the pathogenesis of SLE using unique female mouse models (C57BL/6, MRL+/+ and MRL/lpr) of 6 and 18 weeks with varying degrees of disease progression. Fecal microbiome diversity and composition, gut oxidative stress (OS), barrier function and inflammation, as well as systemic autoimmunity were determined. Interestingly, each mouse strain had distinct bacterial community as revealed by β-diversity. A lower Firmicutes/Bacteroidetes ratio in 6-week-old MRL/lpr mice was observed, evidenced by decrease in *Peptostreptococcaceae* under Firmicutes phylum along with enrichment of *Rikenellaceae* under Bacteroidetes phylum. Additionally, we observed increases in colonic OS [4-hydroxynonenal (HNE)-adducts and HNE-specific immune complexes], permeability changes (lower tight junction protein ZO-2; increased fecal albumin and IgA levels) and inflammatory responses (increased phos-NF-κB, IL-6 and IgG levels) in 18-week-old MRL/lpr mice. These changes were associated with markedly elevated AD markers (antinuclear and anti-smooth muscle antibodies) along with hepatic portal inflammation and severe glomerulonephritis. Notably, antioxidant N-acetylcysteine treatment influenced the microbial composition (decreased *Rikenellaceae*; increased *Akkeransiaceae*, *Erysipelotrichaceae* and *Muribaculaceae*) and attenuated the systemic autoimmunity in MRL/lpr mice. Our data thus show that gut microbiome dysbiosis is associated with increased colonic OS, barrier dysfunction, inflammatory responses and systemic autoimmunity markers. These findings apart from delineating a role for gut microbiome dysbiosis, also support the contribution of gut OS, permeability changes and inflammatory responses in the pathogenesis of ADs.

## Introduction

Autoimmune diseases (ADs), like various complex systemic inflammatory disorders, are driven by combination of genetic, hormonal and environmental factors. Emerging evidence suggest that imbalance in the gut microbiome composition (dysbiosis), an important environmental factor, is associated with multiple ADs, including systemic lupus erythematosus (SLE) and autoimmune hepatitis (AIH) (1–4). Based on accumulating evidence, SLE/AIH patients with active disease have lower bacterial diversity and altered bacterial community composition in the gut (5, 6). Enrichment in *Veillonella* genus, for example, correlates to AIH disease activity, whereas increases in *Ruminococcus gnavus* of the *Lachnospiraceae* family is associated with the SLE disease indices (1, 4, 5). However, the contribution and mechanistic link between microbiome dysbiosis and ADs remain unclear, especially bacterial-host interactions.

Gut barrier function involves the crosstalk and interplay of the gut epithelial and mucosal layers, tight junctional proteins, and the immune system (7, 8). Impaired epithelial integrity and barrier function could be central predisposing factors in a number of ADs (9). Loss of barrier integrity may promote intestinal inflammation and translocation of bacterial components into the circulatory or lymphatic systems, resulting in systemic immune responses, and ultimately leading to SLE/AIH (1, 3). In fact, observed translocation of *Enterococcus gallinarum* and *Lactobacillus reuteri* into non-intestinal tissues suggests that leaky gut could be critical in promoting systemic autoimmune responses (10). Furthermore, mechanisms delineating tight junction regulation of gut permeability and inflammation suggest that posttranslational oxidative modification of proteins could contribute to gut leakiness and eventually disease activity (11–13). Our previous studies using MRL/lpr and MRL+/+ mice have shown that increased oxidative stress along with induction of lipid peroxidation-derived reactive aldehydes (LDRAs) are closely associated with the severity of autoimmune response/disease (14). Extensive ROS can enhance proinflammatory cytokine production and reduce mucus production in the intestinal epithelium (15–17). However, contribution of LDRAs and oxidative modification of proteins in microbiome dysbiosis-mediated mucosal and systemic immune responses remain unclear.

Intestinal microbiome dysbiosis is associated with chronic low-grade inflammation, and also triggers autoreactive T cells (CD4+ and CD8+ T cells) to drive spontaneous autoimmunity in target organs (18–20). The intestinal commensals interact with the host mucosal immune system leading to an imbalance in Treg and Th17 cells (21). Moreover, recent evidence suggests that fecal microbiome transplantation or probiotic supplementation could have beneficial effects in ADs based on evidence from both animal models and human studies (22–28). Furthermore, dietary intervention with retinoic acid or probiotics improves the disease manifestations by modulating microbiome composition, restoring gut barrier function and mucosal Treg-Th17 balance (6, 29). These studies thus suggest that in addition to disturbances in gut microbiome composition (dysbiosis), the interaction between the gut microbiome and host immune system could contribute to the pathogenesis of ADs. Our studies were, thus, designed to test the hypothesis that gut microbiome dysbiosis, colonic oxidative stress and impaired barrier function contribute to leaky gut and systemic inflammatory response, leading to ADs.

Here, we present data showing differential gut microbiome composition in lupus-resistant and lupus-prone mice at different ages, especially evidenced by altered Firmicutes to Bacteroidetes ratio (F/B ratio) and β-diversity. More importantly, changes in the microbiome composition are associated with compromised gut barrier function, serum AD markers, circulating cytokines and infiltration of immune cells in the liver and kidney. Overall, these observations support that dynamic interaction among intestinal microbiota composition, immune cell responses and autoantibody production are highly linked to renal and hepatic inflammation, eventually leading to corresponding ADs, i.e., SLE and AIH.

## Materials and Methods

### Animals and Treatments

Four-week-old female C57BL/6, MRL+/+ and MRL/lpr mice were purchased from the Jackson Laboratory (Bar Harbor, ME) and were maintained under sterile animal facility. All experiments were performed in accordance with protocols approved by the Institutional Animal Care and Use Committee of the University of Texas Medical Branch. In the first set of experiments, 6 and 18 weeks old C57BL/6, MRL+/+ and MRL/lpr mice (n=5 per strain, per time point) were sacrificed and fecal samples were collected from the colon for determining differential microbiome responses. Secondly, to establish the contributory role of oxidative stress in the disease progression, 5-week-old MRL/lpr mice were treated with antioxidant N-acetylcysteine (NAC, 250 mg/kg/day *via* drinking water) (30, 31) or drinking water only for 7 weeks and designated as NAC and control (CON) groups, respectively (n=6 per group). Mice were euthanized and major organs were weighed, frozen in liquid nitrogen, and stored at −80°C for further analyses. Sera obtained from blood samples were stored in small aliquots at −80°C until further analysis.

### Enzyme-Linked Immunosorbent Assays (ELISAs) and Bio-Plex Assay

Autoantibodies [antinuclear antibodies (ANA); anti-smooth muscle antibodies (ASMA)] in the sera were determined using mouse-specific ANA ELISA kit (Alpha Diagnostic Int’l, San Antonio, TX) and mouse ASMA ELISA kit (Cusabio LLC, CA) by following the manufacturer’s instructions. HNE-protein adducts and its specific circulating immune complexes (CICs) in the colon tissues were analyzed according to our earlier published methods (14, 32).

Serum cytokines were determined using Cytokine 17-Plex Mouse ProcartaPlex Panel (Invitrogen, Carlsbad, CA). Samples were assayed and data were collected by Bio-Rad Bio-Plex 200 System. Data were measured as relative fluorescence intensity and then converted to the concentration using the standard curve.

### 16s rDNA Sequencing in Fecal Samples

Fecal samples were collected and stored at -80°C. DNA isolation and 16S rDNA sequencing was performed at the Alkek Center for Metagenomics and Microbiome Research at Baylor College of Medicine. Briefly, total genomic DNA was extracted using the MagAttract PowerSoil Kit (Qiagen, Redwood City, CA). The 16Sv4 region was amplified by PCR and sequenced on the MiSeq platform (Illumina, San Diego, CA) using a 2x250 bp paired-end protocol, yielding paired-end reads that overlap almost completely. For microbiome data analysis, Agile Toolkit for Incisive Microbial Analyses (ATIMA) was used. ATIMA is a stand-alone tool for analyzing and visualizing trends in taxa abundance, alpha diversity, and beta diversity as they relate to sample metadata (33, 34). Quantitative PCR was performed to further verify certain bacterial changes (10, 35).

### Isolation of Lymphocytes From Liver Tissues

Intrahepatic lymphocytes (IHLs) were isolated as previously described (30). In brief, liver was perfused with 10 ml PBS, minced and digested with RPMI 1640 containing 0.05% collagenase IV (Roche, Indianapolis, IN) at 37 °C for 30 min. After digestion, cell suspensions were passed through 70 μm cell strainers, followed by a centrifugation over a 30/70% discontinuous Percoll density gradient (Sigma, St. Louis, MO) at 400 g at room temperature for 30 min. The cells were collected from the interphase, washed, resuspended in complete RPMI 1640, and the total number of IHLs per liver was counted. The relative percentages of immune cell populations were then analyzed by flow cytometry, and the absolute numbers of these lymphocyte subpopulations per liver were calculated according to their percentages and total IHL numbers in each liver.

### Flow Cytometry

The specific antibodies and their corresponding isotype controls were purchased from Biolegend (San Diego, CA) and eBioscience (Waltham, MA). Cells were first incubated with FcγR blocker (CD16/32), followed by fluorochrome-labeled antibodies (Abs). The following Abs were used in combinations: PE-Cy7 anti-mouse CD3, Pacific Blue anti-mouse CD4, APC-Cy7 anti-mouse CD8, APC anti-mouse CD11b, APC-Cy7 anti-mouse CD11c, AF700 anti-mouse CD19 and Percp-cy5.5 anti-mouse CD45R/B220. Flow cytometric analysis was done using an LSRII Fortessa (BD Bioscience, San Jose, CA) and analyzed using FlowJo software 10.0 (TreeStar, Ashland, OR).

### Quantitative Reverse Transcriptase PCR (qRT-PCR) Analysis

Total RNA was extracted from liver tissues using Trizol reagent (Sigma) followed by treatment with Qiagen DNase I (Qiagen). cDNA was synthesized with the iScript reverse transcription supermix (Bio-Rad, Hercules, CA). qRT-PCR was performed using iTaq universal SYBR green supermix kit (Bio-Rad, Hercules, CA) on a Bio-Rad CFX96 real time PCR machine. The mRNA expression of selected genes related to inflammation (TLR4, CD14, MCP1, IL-6, IL-4, IL-10, CXCR9, CXCR10 and TNF-α), inflammasome (NLRP3, caspase1, ASC, IL-1β and IL-18) and oxidative stress (SOD, catalase, OGG1 and iNOS) was determined. Mouse glyceraldehyde 3-phosphate dehydrogenase (GAPDH) was used as the housekeeping gene. The primer sequences for the genes analyzed were according to our previous publication (30, 36). For the quantification of bacterial translocation, DNA was extracted from liver tissues and 100 ng DNA was used for qPCR analysis. The primer sequences for eubacteria were according to previous publication (35).

### Western Blot Analysis

Total colon protein was homogenized in T-PER buffer including 1% protease inhibitor cocktail (Sigma) and protein concentration in the lysates was determined by Pierce™ BCA Protein Assay Kit. Western blot analysis was done for tight junction proteins. Ten µg protein per lane was loaded onto 4-20% Tris-Glycine gel (ThermoFisher scientific, Grand Island, NY) and transferred to PVDF membrane. The membrane was blotted with primary antibodies at 4°C overnight. Antibody detection was accomplished using horseradish peroxidase conjugated secondary antibodies and visualized with ECL. The signal intensity was quantified with Image Studio Lite Ver 5.2 (LI-COR).

### Histological Examination

Liver and kidney tissues were fixed in 10% neutral formalin, dehydrated and paraffin embedded. Paraffin sections (5 µM thick) were stained with hematoxylin-eosin (H&E), and then blindly evaluated for cellular and structural changes in the liver and kidney.

### Fecal IgA and Albumin Assays

Fecal samples were weighed and homogenized in cold PBS (1:10), centrifuged at 10,000 g at 4°C for 10 minutes. The supernatant was used for ELISA analysis of IgA (Invitrogen) and albumin (Bethyl Laboratories, Montgomery, TX) levels according to the manufacturer’s instructions.

### Statistical Analysis

Statistical analysis was performed using GraphPad Prism software 7.0 (GraphPad, La Jolla, CA). One-way analysis of variance (ANOVA) followed by Tukey-Kramer multiple comparison test was done to determine statistical significance between any two groups. The *p* values <0.05 were considered to be statistically significant. *p < 0.05; **p < 0.01. For microbiome data processed *via* ATIMA website, the statistical tests used are Kruskall-Wallis and Mann-Whitney for alpha diversity and taxa abundance, and the PERMANOVA test for beta diversity.

## Results

### Differential Autoimmune Responses in Lupus-Resistant/-Prone Mice

**Figure 1:**
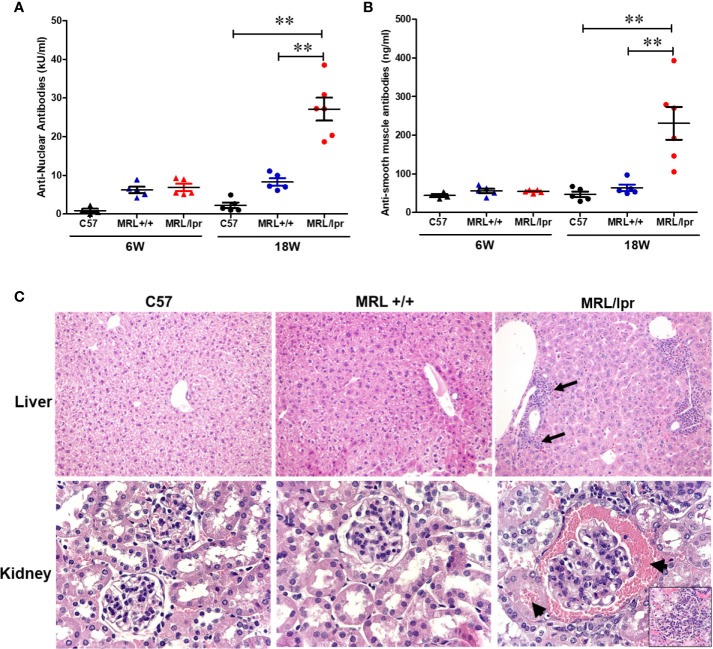
Spontaneous development of SLE- and AIH-like diseases in MRL/lpr mice. Serum ANA levels **(A)** and serum ASMA levels **(B)** in C57BL/6 (C57), MRL+/+ and MRL/lpr mice. Histopathology of liver **(C)** shows marked various peri-portal infiltrates (arrows) in MRL/lpr mice. Renal histopathology (lower panel) shows glomerulus with hypercellularity and red blood cells in Bowman’s spod and adjacent tubule (arrows) in MRL/lpr mice, indicative of acute glomerulonephritis (H&E). n = 5, **p < 0.01.

MRL+/+ mice spontaneously but very slowly develop autoantibodies several months after birth and lupus-like glomerulonephritis (SLE) late in the second year of life (37–39). In contrast, the MRL/lpr mouse strain is a model for more rapid and aggressive onset of SLE due to Fas (CD95) mutation, with the generation of autoantibodies as early as 6 weeks and advanced renal disease (glomerulonephritis) around 16 weeks (40). Female mice were used in this study due to greater propensity to develop ADs in women (41–43). To demonstrate differential autoimmune responses in normal and genetically susceptible mouse strains, we measured serum levels of ANA (a marker for SLE) and ASMA (a marker for AIH) in age-matched female C57BL6, MRL+/+ and MRL/lpr mice. As evidenced from **Figures 1A, B**, ANA and ASMA levels were very low at 6 weeks of age in both MRL+/+ and MRL/lpr mice, but significantly increased in MRL/lpr mice at 18 weeks compared to other two strains. In addition, the increases in the autoantibodies were associated with severe glomerulonephritis and infiltration of various immune cells in the hepatic portal area of 18-week-old MRL/lpr mice (**Figure 1C**). These data indicate systemic autoimmune responses are accompanied by immune cell infiltration in key organs associated with SLE and AIH.

### Hepatic Immune Cell and Pro-Inflammatory Responses in Normal and Lupus-Prone Mice

**Figure 2:**
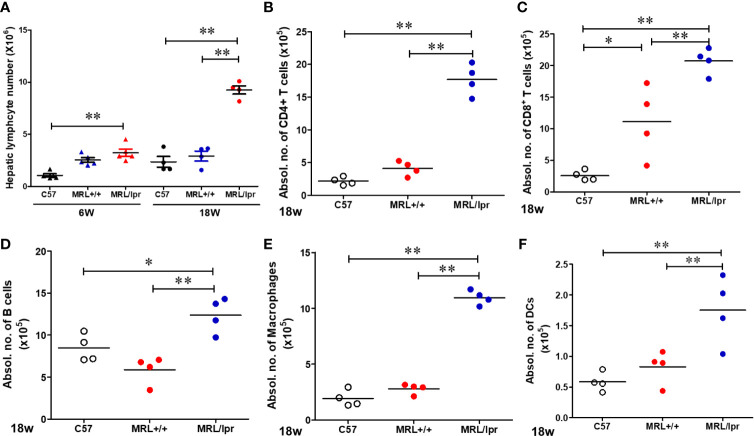
Flow cytometric analysis of hepatic immune cell populations in C57BL/6, MRL+/+ and MRL/lpr mice. **(A)** Hepatic lymphocytes numbers in 6-week and 18-week old mice. **(B–F)** Absolute numbers of CD4+ T cells (CD3+ CD4+ CD8−), CD8+ T cells (CD3+ CD4− CD8+), B cells (CD3− B220+), macrophages (CD11b+ F4/80+) and DCs (CD11B+CD11c+) in the livers of 18-week-old mice. Each symbol represents an individual mouse, and horizontal lines indicate the mean. n = 4 *p < 0.05; **p < 0.01.

**Figure 3:**
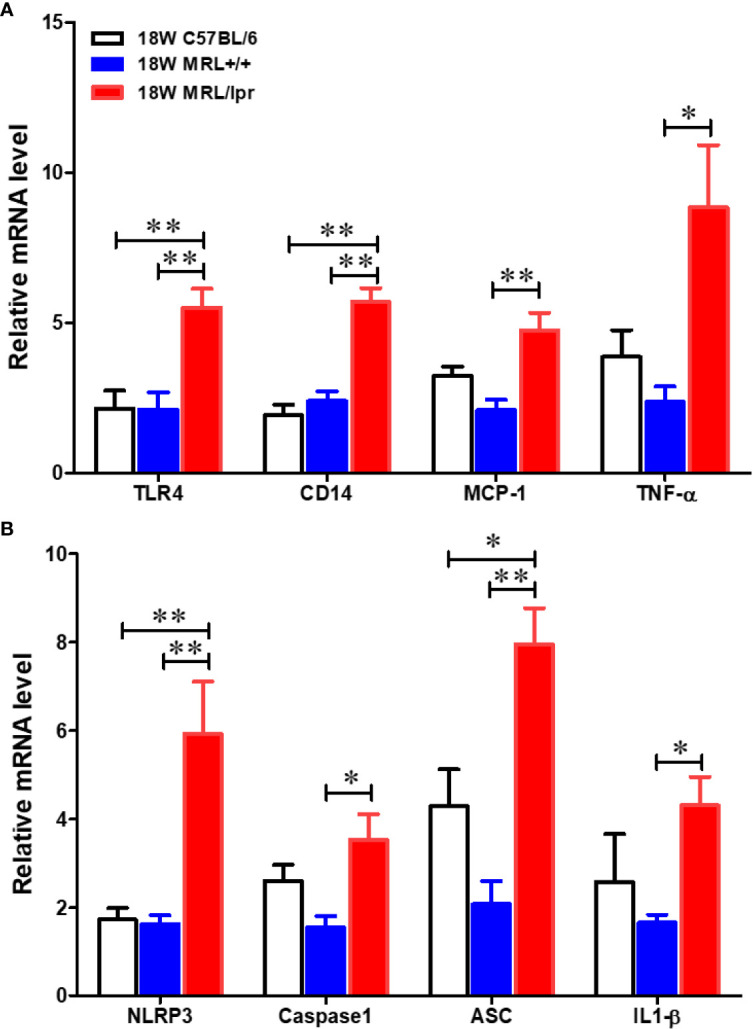
Up-regulation of inflammatory genes in the livers of C57BL/6, MRL+/+ and MRL/lpr mice. Total RNA was extracted from liver tissues and relative mRNA expressions of **(A)** inflammatory markers [Toll like receptor 4 (TLR4), CD14, MCP-1 and TNF-α] and **(B)** inflammasome activation markers (NLRP3, caspase-1, ASC and IL-1β) were determined by RT-PCR. Results are Mean ± SEM. n = 5 *p < 0.05; **p < 0.01.

To address potential cellular involvement in the development of autoimmunity, we measured immune cell populations in the livers of the three mouse strains by FACS. We observed a significant increase of the total hepatic lymphocytes in 18-week-old MRL/lpr mice. Differential analysis showed increased B cells, CD4+ and CD8+ T cells, macrophages as well as dendritic cells in the livers of 18-week-old MRL/lpr mice compared to age-matched MRL+/+ and C57BL6 mice. In contrast, only hepatic CD8+ T cells were elevated in MRL+/+ compared with C57BL6 mice (**Figure 2**). Significantly higher levels of inflammatory markers TLR4 and CD14 were also found in the livers of 18-week-old MRL/lpr mice (**Figure 3A**). Furthermore, hepatic expression of MCP-1 and TNF-α was also increased in 18-week-old MRL/lpr mice (**Figure 3A**) when the disease markers were also highly prevalent. Importantly, inflammasome activation, which is implicated in SLE (30), showed a robust increase in its activation markers (NLRP3, caspase1 and IL-1β) in the livers of 18-week-old MRL/lpr mice (**Figure 3B**). These findings suggest that infiltration of multiple immune cells and increased pro-inflammatory responses may contribute to breakdown of immune tolerance, leading to autoimmune responses.

### Gut Microbiome Compositional Changes in Lupus-Resistant/-Prone Mice at Different Ages

**Figure 4:**
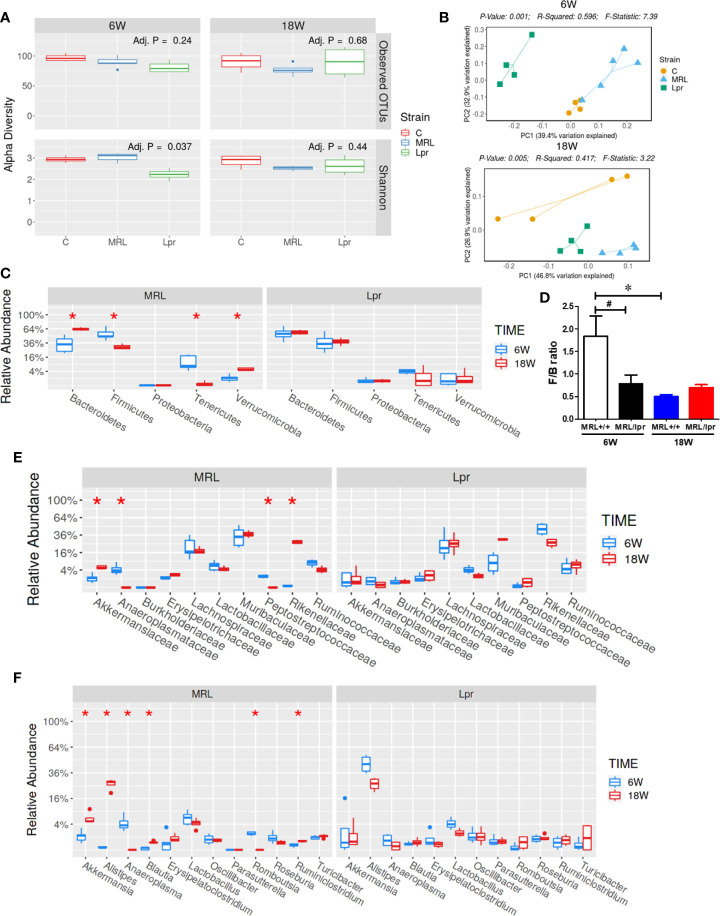
SLE-associated gut microbiome diversity and composition. Fecal pellets from C57BL/6 (C), MRL+/+ (MRL) and MRL/lpr (Lpr) mice were collected and bacterial DNA was isolated and subjected to 16s rDNA sequencing to evaluate the composition of microbiome in each mice. **(A)** The variety of organisms in a community, termed as alpha diversity. **(B)** Different mouse strains showed distinct microbiome pattern at 6 week and 18 weeks of age. **(C)** Age-associated differences in fecal microbiome from MRL+/+ and MRL/lpr mice at phylum level. **(D)** F/B ratio in MRL+/+ and MRL/lpr mice. **(E)** Taxa relative abundance at family level in MRL+/+ and MRL/lpr mice. **(F)** Taxa relative abundance at genus level in MRL+/+ and MRL/lpr mice. Results are mean ± SEM. n = 4 *p < 0.05; ^#^ *p* < 0.1.

**Figure 5:**
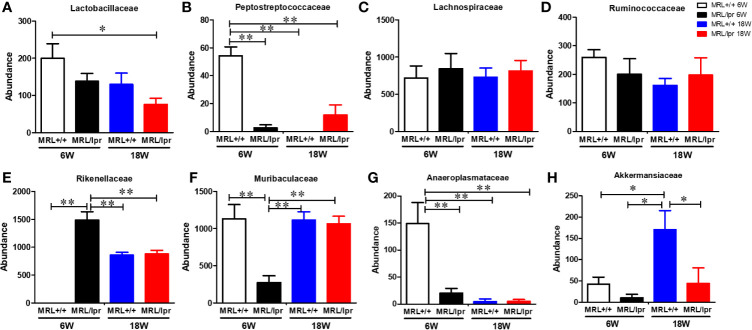
Differential abundance of bacterial families in MRL+/+ and MRL/lpr mice at 6 and 18 weeks of age. **(A)** Abundance of *Lactobacillaceae* under Firmicutes. **(B)** Abundance of *Peptostreptococcaceae* under Firmicutes. **(C)** Abundance of *Lachnospiraceae* under Firmicutes. **(D)** Abundance of *Ruminococcaceae* under Firmicutes. **(E)** Abundance of *Rikenellaceae* under Bacteroidetes **(F)** Abundance of *Muribaculaceae* under Bacteroidetes **(G)** Abundance of *Anaeroplasmataceae* under Tenericutes. **(H)** Abundance of *Akkermansiaceae* under Verrucomicrobia. Results are mean ± SEM. n = 4 *p < 0.05; **p < 0.01.

Gut microbiome composition could have a pivotal role in triggering autoimmunity in susceptible individuals (2). To investigate the involvement of the gut microbiome in SLE, we compared the microbiome composition in C57BL6, MRL+/+ and MRL/lpr mice at different stages in disease development. Fecal samples, collected before disease onset (6 weeks) and at the peak of the disease process in MRL/lpr mice (18 weeks) were subjected to 16S rDNA sequencing for microbiome analysis. Compared to the other two strains, six-week-old MRL/lpr mice had significantly lower alpha diversity as measured by Shannon index but not observed OTU (**Figure 4A**). However, these observations were no longer evident by 18 weeks. These data suggest early differences in bacterial distribution but not overall community richness. Principal coordinates analysis of beta diversity further revealed differential bacterial community composition in each mouse strain at both 6 and 18 weeks derived from weighted UniFrac (**Figure 4B**). There was no significant difference between C57BL/6 and MRL+/+ mice for α-diversity at either time point, and no autoantibodies developed in C57BL/6 mice. Therefore, subsequent comparisons were mainly done between MRL+/+ and MRL/lpr mice at both time points. Dynamic changes in the fecal microbiome composition were observed between 6- and 18-week MRL+/+ mice, evidenced by increased levels of Bacteroidetes and Verrucomicrobia phyla and concomitant reductions in Firmicutes and Tenericutes (**Figure 4C**) at the latter time point. These shifts translated to the family level whereby increases in *Akkermansiaceae* and *Rikenellaceae* accompanied by relative losses of *Anaeroplasmataceae and Peptostreptococcaceae* in MRL+/+ mice over time (**Figure 4E**). At the lower taxonomic genus level, enrichment of *Akkermansia*, *Alistipes, Blautia and Ruminiclostridium* was observed in 18-week compared to 6-week MRL+/+ mice together with reduction in *Anaeroplasma* and *Romboutsia* (**Figure 4F**). Furthermore, the abundance of specific intestinal bacterial strain was also measured by RT-PCR. Increase in *Akkermansia muciniphila* under Verrucomicrobia, while reduction in *Clostridium clostridiiforme* and *Faecalibacterium prausnitzii* under Firmicutes were observed in fecal samples of 18-week-old MRL+/+ mice (**Supplementary Figure 1**). Collectively, we observed dramatic age-related changes in the gut microbiome of MRL+/+ mice, suggesting dynamic microbiome shift happens before the disease progression which could be a potential early event contributing to autoimmune responses. A lower F/B ratio was observed in the 6-week-old MRL/lpr mice (**Figure 4D**), evidenced by decreased abundance in *Peptostreptococcaceae* and *Lactobacillaceae* under Firmicutes phylum (**Figures 5A, B**) and significant increase in abundance of *Rikenellaceae* under Bacteroidetes phylum (**Figure 5E**) as compared to 6-week-old MRL+/+ mice. Furthermore, lower abundance in *Muribaculaceae* and *Anaeroplasmataceae* in 6-week-old MRL/lpr mice than age-matched MRL+/+ mice (**Figures 5F, G**), and an enrichment of *Akkermansiaceae* in 18-week-old MRL+/+ mice were observed (**Figures 5H**). No difference was observed for *Lachnospiraceae* and *Ruminococcaceae* (**Figures 5C, D**) between these two strains at both time points. Taken together, these data provide evidence for an age-related difference in microbiome composition of the lupus-prone mice that might contribute to aberrant gut permeability and also mucosal immune activation.

### Impaired Gut Barrier Function in 18-Week Old Lupus-Prone MRL/lpr Mice

**Figure 6:**
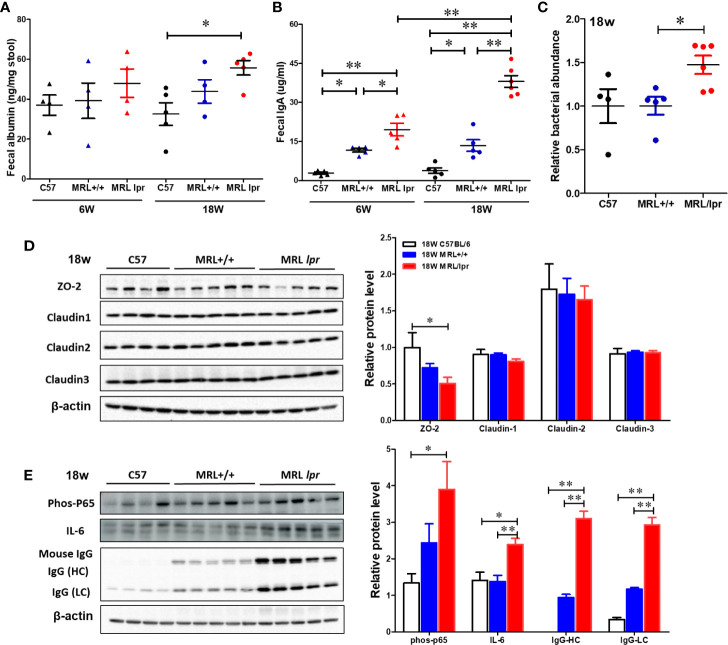
Impaired gut barrier in lupus-prone mice. Fecal albumin and IgA levels were determined by ELISA. Liver DNA was extracted and RT-PCR was performed with Eubacteria primer to detect the bacterial translocation. Colon protein samples were subjected to SDS-PAGE and blotted with specific antibodies. **(A)** Fecal albumin levels. **(B)** Fecal IgA levels **(C)** Bacterial translocation to liver **(D, E)** Western blot for tight junction proteins (ZO-2, Claudin 1-3) and inflammatory markers. Results are mean ± SEM. n = 4 *p < 0.05; **p < 0.01.

**Figure 7:**
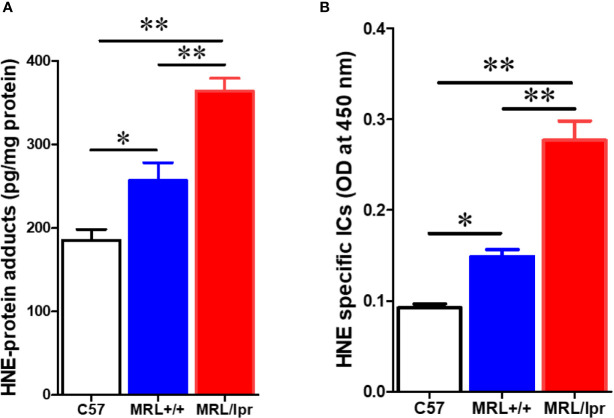
Elevated markers of oxidative stress in the colon of lupus-prone mice. Colon protein samples were subjected to specific ELISA for HNE-protein adducts and HNE-specific ICs. **(A)** HNE-protein adducts. **(B)** HNE-specific ICs. Results are mean ± SEM. n = 4 *p < 0.05; **p < 0.01.

To test whether gut microbiome dysbiosis leads to intestinal inflammation and leakiness, which further contribute to the systemic autoimmune responses, inflammatory markers and tight junction proteins were measured in 18-week samples, when MRL/lpr mice exhibit advanced SLE disease manifestations. Additionally, we also determined the fecal albumin and IgA levels as markers of gut leakage and inflammation. In MRL/lpr mice, altered microbiome composition was associated with increased intestinal permeability, evidenced by increased fecal albumin and IgA levels (**Figures 6A, B**) and decreased gut tight junction protein ZO-2 (**Figure 6D**). In addition, intestinal inflammation was observed with increased colonic phos-NF-κB, IL-6 and IgG levels in 18-week-old MRL/lpr mice (**Figure 6E**). Based on our histological findings and flow cytometry data showing increased hepatic immune cell infiltration and activation, we also determined the bacterial translocation by amplifying the universal eubacteria in the liver tissues. Interestingly, we observed significantly increased eubacteria in the livers of MRL/lpr mice (**Figure 6C**). Considering that protein oxidation or lipid peroxidation has the potential to affect intestinal permeability *in vitro* and *in vivo*, we analyzed HNE-protein adducts and HNE-specific immune complexes (ICs) in the colon. Interestingly, significantly increased levels of both HNE-protein adducts and HNE-specific ICs were observed in the colon of 18-week-old MRL/lpr mice compared to age-matched C57BL/6 or MRL+/+ mice. Moreover, these oxidative stress (OS) markers were markedly increased in MRL+/+ mice compared to C57BL/6 mice (**Figure 7**). Collectively, these data support an association between gut microbiome dysbiosis-associated OS, intestinal permeability and inflammation in the colon of lupus-prone MRL/lpr mice.

### NAC Supplementation Modulates the Gut Microbiome and Lupus Pathogenesis

**Figure 8:**
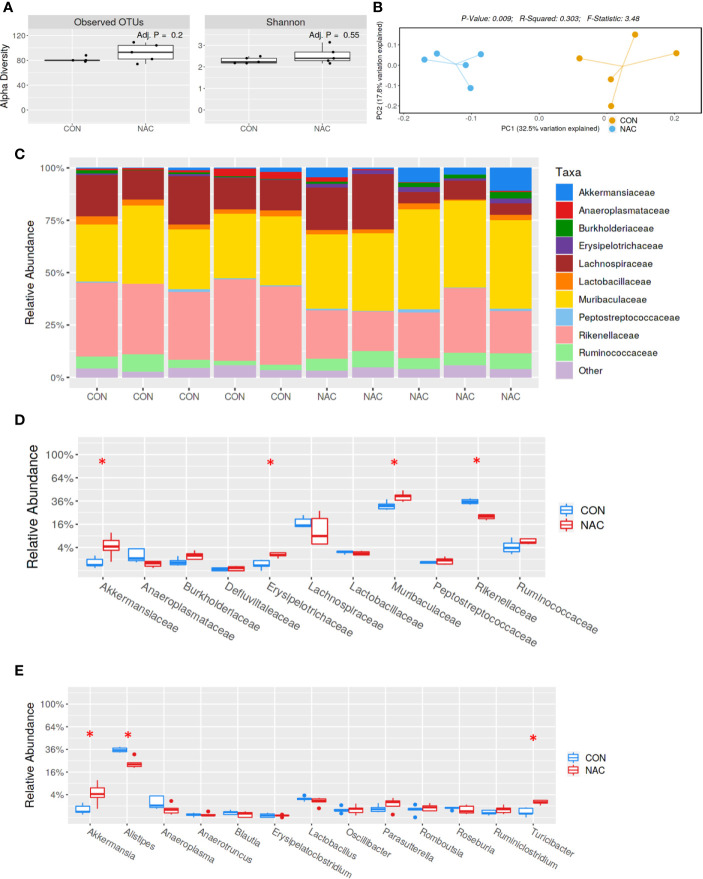
Gut microbiome changes after antioxidant NAC treatment in MRL/lpr mice. Bacterial DNA was isolated from fecal pellets of CON and NAC-treated MRL/lpr mice, and 16s rDNA sequencing was performed to evaluate the microbiome composition in each mice. **(A)** The variety of organisms in a community termed as alpha diversity. **(B)** Different mouse strains showed distinct microbiome pattern. **(C)** Stacked bar plot for composition of common bacterial taxa (>0.1% abundance). **(D)** Taxa relative abundance at family level. **(E)** Taxa relative abundance at genus level. Results are mean ± SEM. n = 5 *p < 0.05.

**Figure 9:**
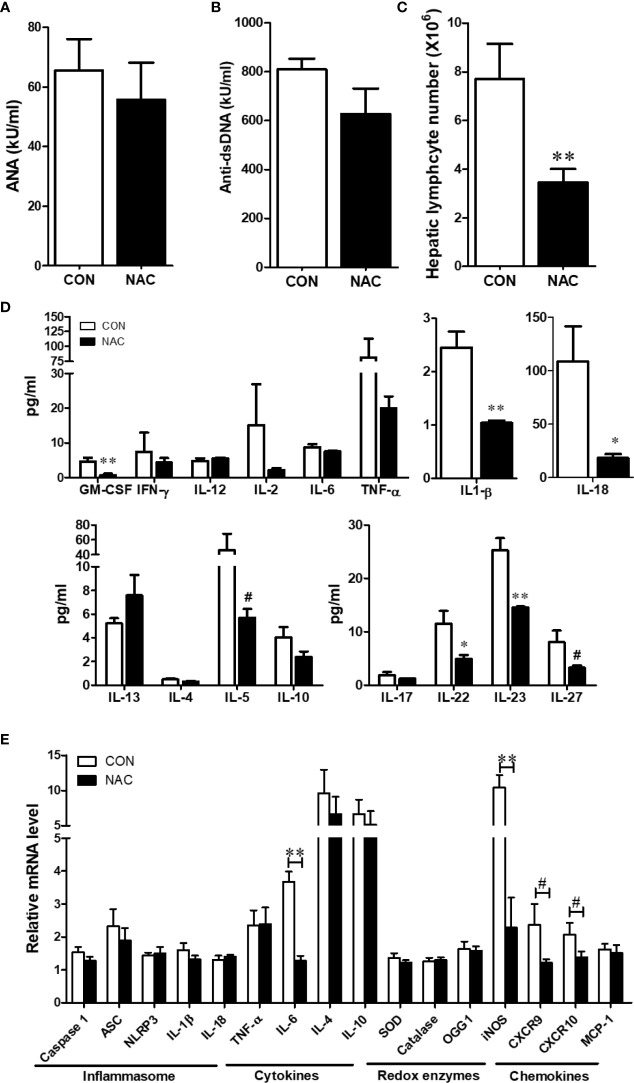
NAC treatment attenuated the auto-inflammatory responses in MRL/lpr mice. Serum ANA **(A)** and anti-dsDNA **(B)** levels in the control and NAC-treated mice. **(C)** Diminution of hepatic lymphocytes following NAC treatment. **(D)** Serum cytokines, measured using a multiplex assay in the control and NAC-treated mice. **(E)** RT-PCR for markers of inflammasome activation, cytokines, redox-related enzymes and chemokines in the control and NAC-treated mice. Results are mean ± SEM. n = 5 *p < 0.05; **p < 0.01, ^#^ *p* < 0.1.

To investigate whether an antioxidant could ameliorate the gut microbiome and pathological changes as well as SLE disease activity, MRL/lpr mice were treated with N-acetylcysteine (NAC), an antioxidant which is a precursor of GSH and detoxifies ROS (31, 44). Although NAC treatment did not alter α-diversity (richness and evenness) (**Figure 8A**), it did lead to distinct gut microbial community changes revealed by β-diversity and bacterial taxonomy at the family level (**Figures 8B, C**) evident from relative increases in bacterial families *Akkermansiaceae*, *Erysipelotrichaceae* and *Muribaculaceae*, and decreased *Rikenellaceae* (**Figure 8D**). At genus level, NAC supplementation led to enrichment in *Akkermansia* and *Turicibacter*, while reduction in *Alistipes* (**Figure 8E**). Even though NAC treatment resulted in a decreasing pattern for both ANA and anti-dsDNA levels in the serum, the differences did not reach statistical significance (**Figures 9A, B**). We also observed significant reduction in hepatic lymphocyte number (**Figure 9C**) and multiple serum cytokines (GM-CSF, IL-1β, IL-18, IL-5, IL-22, IL-23 and IL-27) following NAC treatment (**Figure 9D**). Furthermore, levels of hepatic IL-6, iNOS, CXCR9 and CXCR10 were also significantly attenuated following NAC supplementation in MRL/lpr mice (**Figure 9E**). Overall, our data suggest a beneficial role of NAC for SLE disease attenuation and also provide mechanistic evidence for the involvement of OS in the regulation of mucosal and systemic immune responses.

## Discussion

SLE and AIH are chronic ADs that occur worldwide with a rising prevalence rate (45). These ADs are characterized by loss of tolerance to self-antigens and aberrant immune activation. Recent studies suggest association of gut microbiome dysbiosis with ADs, although it remains to be established how the microbiotia contribute to SLE/AIH pathogenesis. Based on recent evidence, some key perturbations in the gut, including increased OS, changes in permeability (leaky gut) and activation of the mucosal immune system are suggested as few mechanisms that could potentially contribute to the pathogenesis of ADs (2). To better define the role of the gut microbiota and mechanisms involved in disease pathogenesis, in this study, we characterized the gut microbiome and mucosal pathophysiological changes in the progression of systemic autoimmunity using three strains of mice with different manifestations of SLE disease activities.

MRL+/+ mice exhibit a similar autoantibody profile and glomerulonephritis as seen in lupus patients (38, 39). It is well-established that MRL+/+ mice would also develop AIH features, including hepatic immune cell infiltration and cytokine production as seen in response to trichloroethene exposure (30, 46, 47). On the other hand, MRL/lpr mice spontaneously develop accelerated autoimmune responses due to Fas (CD95) mutation (37). Here, we observed that 18-week-old MRL/lpr mice had increased ANA levels and severe glomerulonephritis representing typical SLE disease manifestations, as well as lymphocytic infiltration in the hepatic portal area and elevated ASMA levels indicating prevalence of AIH in these mice. Furthermore, analysis of hepatic immune cell populations revealed significant increases in the absolute numbers for T and B cells in 18-week-old MRL/lpr mice compared to C57BL/6 and MRL+/+ mice. Our data apart from firmly establishing that aged MRL/lpr mice manifest SLE and AIH features, also provide solid support to utilizing our approach of using age-matched different strains of mice with distinct disease activities in exploring the potential contributing factors and mechanisms leading to SLE and AIH.

Based on recent studies, it is becoming clear that altered gut microbiome composition and impaired barrier function are associated with multiple ADs, including SLE/AIH both in patients and murine models (2–4, 48). In fact, evaluation of feces from SLE patients has provided evidence for a lower F/B ratio (49). Here, we also observed a lower F/B ratio in 6-week old MRL/lpr mice compared to age-matched MRL+/+ mice. Interestingly, age-related decrease in F/B ratio was also observed in 18-week-old MRL+/+ mice. Although decreased F/B ratio is not correlated with highly advanced disease activity in 18-week MRL/lpr mice, a lower F/B ratio at 6 weeks could be an important and highly significant contributing factor in promoting early disease onset. Lactobacillus, a known probiotic, exhibits its anti-inflammatory effect in ADs by inducing T regulatory (Treg) cells (50). *Lactobacillus* colonization attenuates SLE by modulating Treg-Th17 balance and gut barrier function (29). An earlier study showed depletion of *Lactobacilli* and increases in *Lachnospiraceae* in 5-week-old young MRL/lpr mice compared to MRL+/+ controls (6). Here, we observed decreasing trend of *Lactobacillaceae* in 18-week-old MRL/lpr mice compared with 6-week-old counterparts, suggesting a prolonged deficiency of *Lactobacillaceae* may consequently result in the generation of mucosal inflammatory responses. It has been reported that *Akkermansia muciniphila* is increased in multiple sclerosis patients and can induce pro-inflammatory responses *in vitro* and *in vivo* (51, 52). Interestingly, in this study, *Akkermansiaceae* was significantly increased in 18-week old MRL+/+ mice compared with 6-week old counterparts. However, MRL/lpr mice had lower level of *Akkermansiaceae* compared to age-matched MRL+/+ mice. The differential responses in the two strains (MRL+/+ vs MRL/lpr) provide a unique opportunity to explain their role in slow vs. aggressive disease development and thus warrant a detailed investigation of their contribution in SLE pathogenesis.

Gut microbial communities can influence host immune responses and epithelium integrity (7, 8). Therefore, we determined the gut barrier integrity and function, as well as inflammatory markers. Our data show that 18-week-old MRL/lpr mice had significantly higher fecal albumin and IgA levels and reduced tight junction protein ZO-2, which can contribute to gut barrier dysfunction. In fact, these observations are also supported by findings in SLE patients. Azzouz et al. (1) reported an outgrowth of *Ruminococcus gnavus* under the *Lachnospiraceae* family, and impaired barrier function evidenced by increased fecal IgA, IgM, IgG and calprotectin levels in SLE active patients. Mucosal IgA level is highly associated with commensal exposure and increased fecal IgA in SLE patients suggests impaired intestinal barrier functions (1, 53). One consequence of impaired intestinal barrier function could be systemic translocation of bacteria or bacterial products due to gut leakiness, and endotoxemia has been reported in chronic inflammatory diseases, including SLE (29, 54). This is further substantiated by findings on the translocation of *Enterococcus gallinarum* from intestine to systemic tissues (10), which can trigger inflammatory response, autoantibody production and autoimmune disease manifestations in genetically predisposed individuals. The fact that we observed significantly increased eubacteria levels in the liver tissues of 18-week MRL/lpr mice suggests that bacteria or its genetic material (DNA) may translocate to the liver, generating inflammatory response and eventually leading to hepatic autoimmune responses. Further support to this was evidenced by our observation of significantly increased level of inflammatory cytokines in the serum of 18-week MRL/lpr mice, indicating compromised barrier function and likely activation of mucosal immune system resulting in systemic inflammation. Our data thus provide evidence that gut microbiome dysbiosis could be a potential mechanism leading to autoimmunity *via* epithelial barrier disruption, release of bacteria/bacterial products and consequent systemic immune dysregulation.

Oxidative stress is considered a contributing factor in initiating apoptosis and inflammation leading to ADs (30, 55–57). Our previous studies have shown that OS, including increased MDA-/HNE-protein adducts in the liver and kidney of MRL/lpr mice, is associated with autoimmune responses (14). Here, we provide evidence for significantly increased HNE-protein adducts and HNE-specific ICs in the colon tissue, suggesting that gut microbiome dysbiosis and subsequent interaction with host tissue can change the gut redox potential, which can result in altered gut immune response and barrier function. Loss in intestinal barrier function can facilitate translocation of microbial components and inflammatory mediators, thus contribute to systemic ADs. Since antioxidants can restore normal gut redox status and extra-intestinal redox potential (58–60), we treated MRL/lpr mice with NAC, a ROS scavenger. Ironically, we observed an ameliorated autoimmune response, as evident from decreased pattern for both ANA and anti-dsDNA autoantibodies and suppression of hepatic inflammation after NAC supplementation in MRL/lpr mice. Besides downregulation of hepatic inflammasome activation markers, NAC also reduced the levels of cytokines in the circulation, suggesting the potential of NAC to serve not only as an antioxidant, but also in generating anti-inflammatory response. More importantly, NAC supplementation altered the gut microbiome composition, including increases in *Akkermansiaceae.* Previous work has established that *A. muciniphila* is diminished in lupus-prone NZB/W F1 mice as disease progressed, and expansion of *A. muciniphila* exerts beneficial impact on host metabolism and suppresses autoimmunity (4, 61, 62). In addition, we also observed a reduction of *Rikenellaceae* family after NAC treatment, which is consistent with higher abundance of *Rikenellaceae* family associated with more severe SLE-like disease in SNF1 mice (63). The modification of the gut microbiota by NAC supplementation could be an important mechanistic factor for the beneficial role of antioxidants in regulating gut microenvironment and thereby preventing autoimmunity. Whether NAC exerts anti-inflammatory effects through microbial modulation or by acting directly on the mucosal lymphocytes is an interesting and challenging proposition, and deserves an extensive evaluation to clarify the actual role of OS on gut microbiome and/or mucosal immunity.

In summary, the data in this study suggest that microbiome-host interactions can contribute to autoimmune responses. Moreover, our findings on OS appear to be an important and mechanistically critical event associated with microbiome dysbiosis and barrier dysfunction in ADs. Delineating how intestinal microbial dysbiosis and host dysregulation happen will benefit the understanding of the pathophysiology of ADs. Our study addresses this knowledge gap and reveals that manipulation of intestinal microenvironment redox status could lead to devising therapeutic strategies for ADs. Our studies provide solid platform to further explore and exploit the dynamics of microbiome changes and gut barrier dysfunction in causing mucosal immune dysregulation, especially imbalance between Treg and Th17 cells, and ultimately their contribution to systemic immune response leading to devastating diseases like SLE and AIH.

## Data Availability Statement

The original contributions presented in the study are publicly available. This data can be found here: NCBI repository, PRJNA706579.

## Ethics Statement

The animal study was reviewed and approved by UTMB IACUC.

## Author Contributions

HW, NB, GW, YL, and XD performed the experiments. MK and HW designed the experiments. All authors contributed to the article and approved the submitted version.

## Funding

This work was supported by RO1 grants [ES016302 and ES026887] from the National Institute of Environmental Health Sciences (NIEHS), NIH, and UTMB Institute for Human Infections and Immunity (IHII). The contents are solely the responsibility of the authors and do not necessarily represent the official views of the NIEHS, NIH.

## Conflict of Interest

The authors declare that the research was conducted in the absence of any commercial or financial relationships that could be construed as a potential conflict of interest.

## Supplementary Material

Supplementary Figure 1 Dynamic changes in gut bacterial compositions in MRL+/+ and MRL/lpr mice at 6 and 18 weeks of age. Real-time quantitative PCR analysis was performed by amplifying fecal DNA with primers specific for **(A)** *Akkermansia muciniphila*, **(B)** *Lactobacillus acidophilus*, **(C)** *Faecalibacterium prausnitzii*, **(D)** *Clostridium butyricum* and **(E)** *Clostridium clostridiiforme.* Results are mean ± SEM. n = 5 **p* < 0.05; ***p* < 0.01
