# Supplementary figures and images for: Aberrant Gut Microbiome Contributes to Intestinal Oxidative Stress, Barrier Dysfunction, Inflammation and Systemic Autoimmune Responses in MRL/lpr Mice
Source: Front Immunol. 2021 Apr 12;12:651191. doi: 10.3389/fimmu.2021.651191 (PMC8071869; doi:10.3389/fimmu.2021.651191)

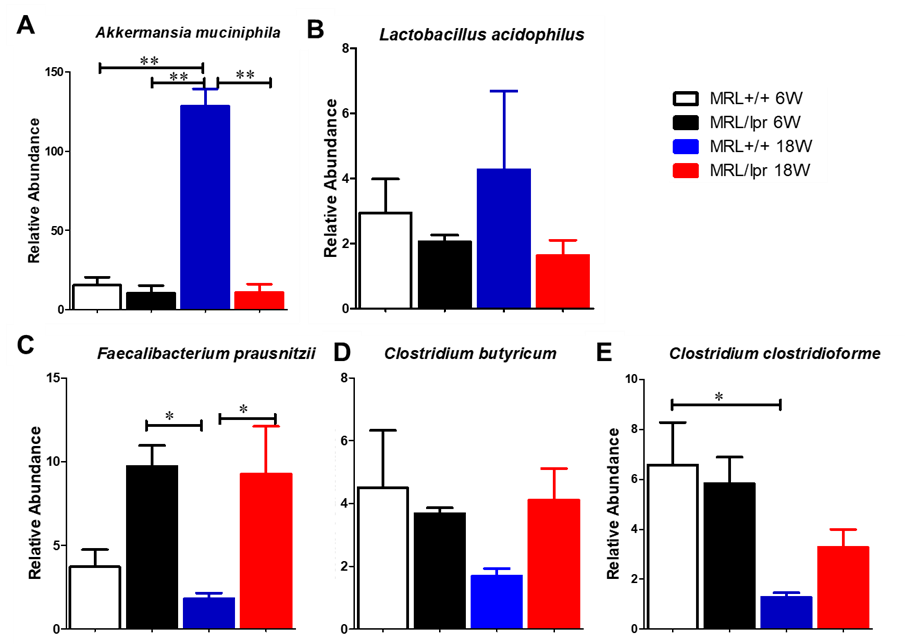

Supplement: Supplementary Figure 1 — Dynamic changes in gut bacterial compositions in MRL+/+ and MRL/lpr mice at 6 and 18 weeks of age. Real-time quantitative PCR analysis was performed by amplifying fecal DNA with primers specific for (A) Akkermansia muciniphila, (B) Lactobacillus acidophilus, (C) Faecalibacterium prausnitzii, (D) Clostridium butyricum and (E) Clostridium clostridiiforme. Results are mean ± SEM. n = 5 *p < 0.05; **p < 0.01 [file Image_1.tif]
